# Grease Waste as a Reservoir of Lipase-Producing Yeast and Description of *Limtongella siamensis* gen. nov., sp. nov

**DOI:** 10.3390/microorganisms8010027

**Published:** 2019-12-22

**Authors:** Varunya Sakpuntoon, Jirameth Angchuan, Wanatchaporn Boontham, Pannida Khunnamwong, Chanita Boonmak, Nantana Srisuk

**Affiliations:** Department of Microbiology, Faculty of Science, Kasetsart University, Bangkok 10900, Thailand; varunya1994@hotmail.com (V.S.); jirameth.a@ku.th (J.A.); wanatchabhorn@gmail.com (W.B.); pannida_minn@hotmail.com (P.K.); fscictb@ku.ac.th (C.B.)

**Keywords:** *Limtongella* gen. nov., *Limtongellasiamensis* sp. nov., lipase producing yeast, oleaginous yeast

## Abstract

A total of 175 yeast isolates were obtained from grease samples. Based on the D1/D2 region of the large subunit (LSU) ribosomal RNA (rRNA) gene analysis, 150 yeast isolates were identified as belonging to 36 described yeast species, whereas 25 isolates required more analysis. Among the described species, *Rhodotorula mucilaginosa* was the only Basidiomycetous yeast, whereas 149 isolates were identified as belonging to 35 described species of 15 genera in the phylum Ascomycota, and *Candida tropicalis* was the most abundant species. A study of lipase production indicated that strain DMKU-JMGT1-45 showed volumetric activity of 38.89 ± 9.62 and 155.56 ± 14.70 U/mL when grown in yeast extract malt extract (YM) and YM supplemented with 1% olive oil, respectively. In addition, this strain intracellularly accumulated lipid, of which the fatty acid profile revealed the major fatty acids to be 39.9% oleic acid (C18:1), 27.61% palmitoleic acid (C16:1) and 14.97% palmitic acid (C16:0). A phylogenetic analysis of the combined multi-locus gene sequences showed that the strains DMKU-JMGT1-45^T^ and DMKU-JMGT4-14 formed a well-separated lineage and could not be assigned to any of the currently recognized genera of the Saccharomycetales. *Limtongella siamensis* gen. nov., sp. nov. is therefore proposed to accommodate these two strains as members of the order Saccharomycetales.

## 1. Introduction

Yeast is a heterotrophic microorganism that uses organic compounds as energy and carbon sources. Many yeasts have been discovered in waste that contain a high organic content. *Cyberlindnera samutprakarnensis* was reported as a novel ascomycetous yeast that was isolated from the wastewater of a cosmetic factory in Samutprakarn province, Thailand [1], while a novel genus of ascomycetous yeast, *Allodekkera sacchari*, was obtained from the waste of a sugar production plant in Nakhon Ratchasima province, Thailand [2]. The continuous reports of new yeast species suggest that organic waste in Thailand may be an attractive source to investigate yeast communities.

A grease trap is a chamber for separating fat from food waste. It consists of organic compounds and a high lipid content. For this reason, a sample from a grease trap was considered a good source to investigate yeast communities and also to discover a promising yeast that possesses the capability to produce lipase, which is an important enzyme in broad industrial applications. Lipase-producing yeasts have been continuously discovered and reported, for example *Candida albicans*, *Candida cylindracea, Candida parapsilosis, Candida tropicalis*, *Saccharomyces cerevisiae,* and *Yarrowia lipolytica* [3]. However, *Candida rugosa* is the most frequently used yeast for the production of commercial lipase. A novel cold active lipase, of which activity was retained by 70% at 10 °C as compared to the maximum value at 35 °C, was produced by *Pichia lynferdii* Y-7723 [4]. A halophilic yeast, *Debaryomyces hansenii* YLL29, isolated from dry-salted olives, showed 7.44 U/mL lipolytic activity [5]. Additionally, there has also been a report of lipase production in novel and undescribed yeasts. *Limtongozyma siamensis* DMKU-WBL1-3, a novel yeast genus isolated from a grease trap, was also shown to produce lipase [6], whereas *Cryptococcus* sp. S-2, an undescribed yeast species isolated from air, was reported to produce a lipase with 65.7 U/mL lipase activity at pH 7.0 and 37 °C, and the enzyme was shown to be stable between pH 5.0 and 9.0 and at temperatures up to 50 °C [7].

Beside lipase-producing ability, lipid accumulation is also a parallel character that has been found in lipase-producing yeasts [8]. Oleaginous yeasts or oily yeasts are yeast species that accumulate lipids in the range of 20%–70% of their cell dry biomass when cultivated under optimized conditions, while less than 10% of their cell dry biomass is accumulated in non-oleaginous yeasts [9]. The well-known typical oleaginous yeasts are in the genera *Candida*, *Cryptococcus*, *Rhodotorula*, *Rhodosporidium* and *Yarrowia* [10]. The presence of lipases in environment enables many yeasts to utilize lipids or other compounds with ester bonds as carbon sources. A triglyceride is a molecule that cannot pass through the cell membrane but has to be degraded into glycerol and free fatty acid prior to absorption [11]. The degradation process requires the activity of a lipolytic enzyme that is secreted into an environment where lipids are hydrolyzed. This may be the reason why many lipase-producing microorganisms have been isolated from lipid-rich environments, such as waste from the oil or fat industry [11]. Aside from being able to accumulate a high lipid content, various oleaginous yeasts also have the potential to present remarkable growth and accumulate lipids on waste and by-products of the agro-industry, such as sugar-enriched wastes or the hydrolysates of various products [12]. Thus, oleaginous yeasts have received great attention due to their potential for biotechnological applications [8]. Oleaginous yeasts usually accumulate intracellular lipids in an N-limited condition [13]. Under this condition, the excess carbon turns into fatty acid and is stored in the form of large lipid droplets (LD) in the cellular compartment. The fatty acid composition of a yeast lipids depends on many factors including species, environmental growth conditions, substrate used, and medium components. The general ratio of lipid composition was found to be C18:1 (oleic acid) > C16:0 (palmitic acid)> C18:2 (linoleic acid) = C18:0 (stearic acid), and C18:1 is one of the three main components in high-acid oil-biodiesel [14] Using an oleaginous yeast as a source of lipid production for biodiesel has several advantages compared to other oil sources, such as its high growth rate, its lesser requirement of space, and the fact that its oil production does not depend on climate change [15]. Hence, lipid production by oleaginous yeasts is considered a great alternative oil source for biodiesel production.

During the course of the yeast community study in the grease trap, the strains DMKU-JMGT1-45^T^ and DMKU-JMGT4-14 were found. The multiple genes analysis showed that the two strains were identical but could not be placed onto any known taxonomic position. The closest species of both strains was *Candida incommunis*, which showed a weak relationship with the genus *Yarrowia*, which was classified into the family Trichomonascaceae [16]. Until now, 12 genera with published names have been recognized as members of the family Trichomonascaceae: *Blastobotrys* [17], *Deakozyma* [18], *Diddensiella* [19], *Groenewaldozyma* [20], *Middelhovenomyces* [18], *Nadsonia* [21], *Spencermartinsiella* [22], *Starmerella* [23], *Sugiyamaella* [24], *Wickerhamiella* [25], *Yarrowia* [26], and *Zygoascus* [27]. However, the strains DMKU-JMGT1-45^T^ and DMKU-JMGT4-14 are unrelated to any described genera. We therefore propose the genus *Limtongella* gen. nov. to accommodate the novel species *Limtongella siamensis*. Additionally, we present the potential of lipase production with the preliminary experiment, as well as the intracellular lipid accumulation of the strain DMKU-JMGT1-45.

## 2. Materials and Methods

### 2.1. Strains and Isolation Procedures

Grease samples were collected from grease traps in Kasetsart University, Thailand. Five milliliters of the sample was suspended in 45 mL of sterile water in a 250 mL Erlenmeyer flask and shaken on a rotary shaker at 30 ± 2 °C for 1 h to detach the cells. The samples were serially diluted, and then 100 µL of the diluted sample were spread onto yeast extract peptone dextrose (YPD) agar supplemented with 0.025% sodium propionate and 0.02% chloramphenicol to prevent filamentous fungal and bacterial growth, respectively. The culture was incubated at 30 ± 2 °C until colonies appeared. Strains that possessed different colony morphologies were collected and cross streaked on YPD agar until the pure culture was obtained. For preservation, a single colony was cultivated in a yeast extract malt extract (YM) broth for 18 h. After incubation, the yeast culture was centrifuged, and cell pellets were washed and resuspended with a fresh YM medium. After that, the purified yeast was preserved in a YM broth supplemented with sterile 30% (*v*/*v*) glycerol in a freezer at −80 °C. The new discovered species has also been kept at China General Microbiological Culture Collection Center (CGMCC), CGMCC 2.680; NITE Biological Resource Center (NBRC), NBRC 114140; Portuguese Yeast Culture Collection (PYCC), PYCC 8358, and Thailand Bioresource Research Center (TBRC), TBRC 10242. 

### 2.2. Screening of Lipase Production on Tween 80 Agar

Lipase production was estimated on Tween 80 agar. The yeast isolate was activated in the YM broth for 18 h. Then, each isolate was pointed onto Tween 80 agar. The petri dishes were incubated for 5 days. The lipase activity index (LI) was calculated as follows:

(1) $$\mathrm{Lipase}\;\mathrm{activity}\;\mathrm{index}\;\left(\mathrm{LI}\right)=\frac{\mathrm{Turbid}\;\mathrm{zone}\;\mathrm{diameter}\;\left(\mathrm{mm}\right)}{\mathrm{Colony}\;\mathrm{diameter}\;\left(\mathrm{mm}\right)}$$

### 2.3. Study of Lipase Production in Liquid Medium

The yeast inoculum was cultured in the YM broth and incubated at 30 ± 2 °C for 18 h on a rotary shaker at 170 rpm and was transferred to 50 mL of the YM broth and the YM broth with 1% olive oil in a 250 mL Erlenmeyer flask for 5 days under the same culture conditions. At the end of incubation, the culture was centrifuged at 8200× *g*, 4 °C. A cell-free supernatant was used as crude enzyme for a quantitative analysis of lipase production, which was estimated, on the basis of olive oil hydrolysis, via the titrimetric method [28]. One milliliter of cell-free supernatant was mixed with assay substrate containing 10 mL of 10% homogenized olive oil in 10% gum acacia, 2 mL of a 0.6% CaCl_2_ solution, and 5 mL of a phosphate buffer. The mixture was incubated on a rotary shaker at 200 rpm and 30 °C for 1 h. To stop the reaction, 20 mL of a alcohol:acetone (1:1) mixture was added. The released fatty acids were titrated against 0.1N NaOH by using phenolphthalein as an indicator. The end point was the appearance of a pink color. A lipase activity unit is defined as the amount of enzyme that releases 1 mmol of fatty acids in 1 min under specified assay conditions.

### 2.4. Examination of Intracellular Lipid Accumulation

Lipid accumulation was investigated by Nile red staining under a fluorescence microscope. Yeast was cultivated in a nitrogen-limited medium II broth containing 70 g/L of glucose as a sole carbon source, 0.75 g/L of yeast extract, 0.55 g/L of (NH_4_)_2_SO_4_, 0.4g/L of KH_2_PO_4_ and 2g/L of MgSO_4_, and incubated on a rotary shaker at 150 rpm and 28 °C for 72 h. Lipid accumulation was investigated by staining cells in 40 μL of a culture broth with 10 μL of a Nile red solution (10 μg/L) [29]. Yeast cells were observed with a fluorescence microscope, under which the lipid content showed a strong yellow–gold emission. Fatty acids composition was analyzed by using the Sherlock Microbial Identification System (MIDI) version 6.1 at the Biodiversity Research Centre, Thailand Institute of Scientific and Technological Research (TISTR).

### 2.5. Phenotypic Characterization of Yeast

The morphology, biochemistry and physiology of the strain DMKU-JMGT1-45^T^ were characterized according to the standard methods described by Yarrow [30]. Morphological studies were performed in a YM broth and YM agar at 25 °C for 3 days. After incubation, cell morphology was studied by using a phase-contrast microscope. A study of pseudohyphae formation was performed via slide culture on potato dextrose agar (PDA) at 25 °C. Growth at various temperatures was determined by cultivation yeast on YM agar incubated at 15, 25, 30, 35, 37, 40 and 45 °C. Ascospore formation was examined for individual strains, and the culture was mixed at weekly intervals for three months on YM agar, YPD agar, PDA agar, corn meal agar (CMA), 5% malt extract agar, Gorodkowa agar, and Fowell’s acetate agar [31] at 25 °C. The assimilation of carbon and nitrogen compounds was investigated in a liquid medium.

### 2.6. Molecular Genetic Characterization of Yeast

For molecular taxonomic identification, genomic DNA was extracted from yeast cells grown in the YM broth at the exponential phase. The D1/D2 domain of the large subunit (LSU) rRNA gene was used to identify all yeast isolates with NL1/NL4 primers [32]. For the strain DMKU-JMGT1-45^T^, small subunit rRNA (SSU), large subunit rRNA (LSU), internal transcribed spacer (ITS), translation elongation factor-1α (EF-1α), RNA polymerase II, subunits 1 (RPB1) and 2 (RPB2) genes were amplified with the primer pairs SSU1f/SSU4r, SSU3f/SSU2r, LSU3f/LSU4r, LSU5f/LSU2r [33], NS7A/NL5A [34], YTEF-1F/YTEF-6AR, EF1-1577F/YTEF-6AR, YRPB1-2F/YRPB1-12R, YRPB2-2F/YRPB2-8R and YRPB2-4F/YRPB2-6FR [18], respectively. PCR products were confirmed by agarose gel electrophoresis and purified with a FavorPrep^TM^ Gel/PCR Purification Mini Kit (Favorgen, Austria). The purified PCR product was sent for DNA sequencing at First BASE Laboratories, Malaysia. The sequences were assembled and aligned with the BioEdit version 7.0.5.3 program [35]. Then, the sequences were compared with the GenBank database (http://www.ncbi.nlm.nih.gov/) using BLASTN search. The phylogenetic tree was constructed with the MEGA version 7.0.26 program [36], and the *Schizosaccharomyces pombe* CBS 356^T^ from Central Bureau voor Schimmelcultures, Baarn, The Netherlands was used as the outgroup. The evolutionary distance was calculated from the general time reversible (GTR) model for the maximum likelihood (ML) analysis. A bootstrap analysis for the estimation of confidence levels of the clades was performed on 1000 bootstrap replications [37], only values greater than 50% were shown. Reference sequences were retrieved from GenBank under the accession numbers indicated in the tree.

## 3. Results

### 3.1. Yeast Isolation and Communities of Yeast in Grease Traps

**Figure 1:**
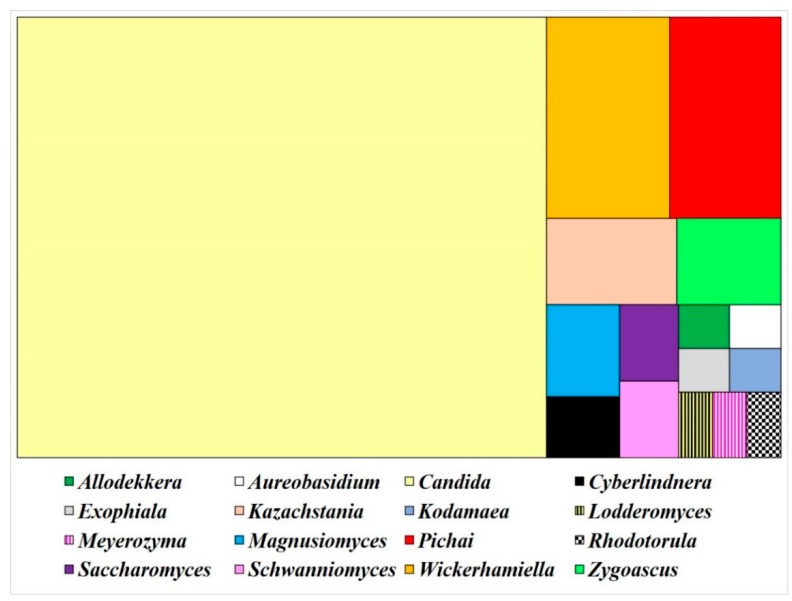
Proportion of yeast genera discovered in grease traps.

Fourteen grease samples were collected from grease traps in eight canteens in Kasetsart University, and 175 yeast isolates were obtained. The D1/D2 domain sequence analysis revealed that 150 isolates (85.71% of total) were identified as 36 described yeast species, whereas 25 isolates (14.29% of total) showed a difference of nucleotide substitution greater than 1% in the D1/D2 domain of the LSU rRNA gene when compared with their closest species. These isolates required more analysis to indicate whether they were undescribed or described yeasts. Surprisingly, *Rhodotorula mucilaginosa* was the only basidiomycetous yeast found in this study, and 149 isolates (85.14% of total) were identified to be 35 species of 15 genera including *Allodekkera, Aureobasidium, Candida* (in *Debaryomyces, Saccharomyces, Pichia, Phaffomyces, Trichomonas, Metschnikowia* and *insertae sedis* clade), *Cyberlindnera, Exophiala, Kazachstania, Kodamaea, Lodderomyces, Magnusiomyces, Meyerozyma, Pichai, Saccharomyces, Schwanniomyces, Wickerhamiella* and *Zygoascus* of the phylum Ascomycota. (Table 1). The results suggest that the majority of yeast species found in the grease traps belong to the phylum Ascomycota, and the yeast population was mainly represented as the genus *Candida* (69.33% of described yeast species), with *Candida tropicalis* being the most abundant yeast species from the entire yeast population (31 isolates and equivalent to 20.67% of described yeast species). The proportion of yeast genera in grease trap samples is shown in Figure 1.

**Figure 2:**
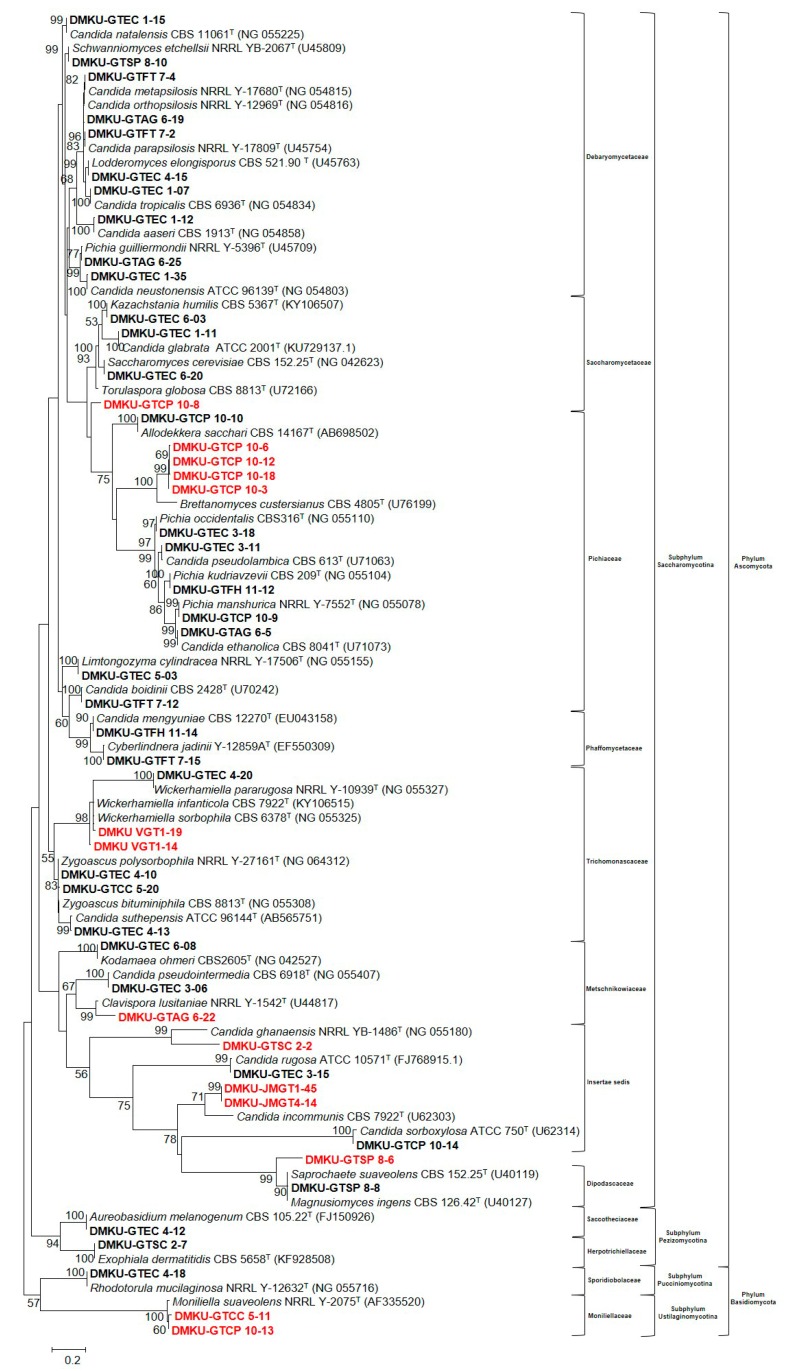
Phylogenetic relationships among the sequences of the D1/D2 domain of the large subunit (LSU) rRNA gene of representative yeast species from grease traps in the phylum Ascomycota, the phylum Basidiomycota, and their closely related yeast sequences retrieved from the GenBank database, as calculated using maximum likelihood method (GTR model). Undescribed species are presented in red color and the isolated yeast are presented in bold letters. Numbers on branches represent the bootstrap percentage (>50%) from 1000 random replications.

The phylogenetic relationships among the obtained yeast in the phyla Ascomycota and Basidiomycota and their closely related species are shown in Figure 2. It is obviously shown that 25 isolates of total yeasts from grease samples appeared to be undescribed yeast species. However, they were also subjected to screening for lipase-producing ability.

### 3.2. Lipase Production and Lipid Accumulation

A rapid method used for investigating of lipase production was done on a Tween 80 medium by measuring the occurrence of the turbid zone, which is the calcium salt of the fatty acids released as a product of lipid hydrolysis. A total of 175 yeast isolates were subjected to screening for lipase production on Tween 80 agar after five days of cultivation. Table S1 shows that 61 isolates produced lipase with a range of 1.1–5.6 on the lipase activity index (LI), although some isolates showed lipase activity irrespective of the incubation period. Among the 61 isolates determined, the strain DMKU-JMGT1-45 showed the highest lipase activity with 5.6 LI value. The strain was then subjected to quantitative analysis for lipase activity. The result revealed that the strain DMKU-JMGT1-45 possessed lipase activity of 38.89 ± 9.62 and 155.56 ± 14.70 U/mL in the YM medium and the YM plus 1% olive oil medium, respectively, when cultivated at 30 °C and 170 rpm for five days. This result suggests that the strain DMKU-JMGT1-45 has potential for lipase production in a medium with oil supplementation; however, the strain was able to produce lipase even with oil omission. It is therefore of interest to investigate the lipase production and enzyme characteristics of this yeast strain in order to obtain information that hints at broader applications of the enzyme. Moreover, yeast with lipase production ability may be related, as previously mentioned, to the ability to accumulate lipids. A study of lipid accumulation and lipid profile was also reported in the strain DMKU-JMGT1-45.

**Figure 3:**
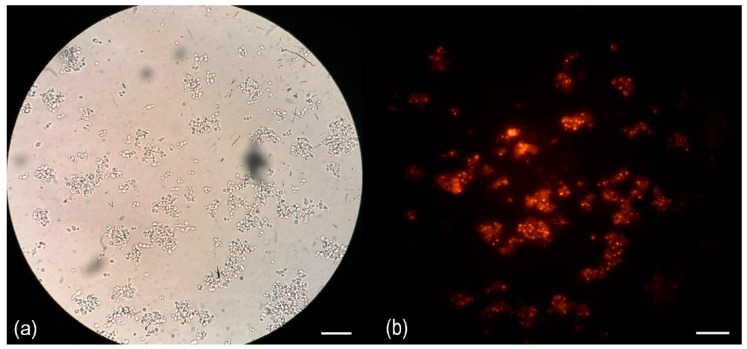
Photomicrographs of the Nile red-stained cells of *Limtongella siamensis* DMKU-JMGT1-45 diluted two folds from a cell density og 1.7 × 10^9^ cell/mL when cultivated in a nitrogen-limited medium II broth at 150 rpm and 28 °C for 72 h. Bar, 20 μm: (**a**) bright-field image; (**b**) fluorescence image.

The qualitative analysis of yeast cellular lipid content that used Nile red staining revealed that the strain DMKU-JMGT1-45 contained lipid granules inside the cell (Figure 3). The fatty acid profile of the strain DMKU-JMGT1-45 cellular lipid was determined. The fatty acid contents in the strain DMKU-JMGT1-45 are shown in Table 2, while the three main fatty acid contents were found to be summed feature 8 (C18:1 Cis 9 (ω 9)/ C18:1 (ω 8), C16:1 Cis 9 (ω 7), and C16:0 with 39.90 %, 27.61% and 14.97% of the total fatty acids, respectively. Oleic acid (C18:1) is one of the main fatty acid components of biodiesel. Thus, oleaginous yeast is considered as an alternative oil source for biodiesel production. However, fatty acid composition or even the type of fatty acid—such as saturated fatty acids (SFA) or unsaturated fatty acids (UFA)—affect the physical properties of biodiesel. For example, those fatty acids that contain little or no unsaturation were found to be more suitable for biodiesel production than polyunsaturated fatty acids. Unsaturated fatty acids with four double bonds or more become limiting to biodiesel production due to their susceptibility to oxidation [38]. Therefore, an optimum ratio of SFA to UFA is necessary for biodiesel use. However, the technical specifications for biodiesel depend on the country or region.

As described above, the strain DMKU-JMGT1-45 obviously possesses both a lipase-producing ability and lipid accumulation. It was therefore of interest to describe the strain DMKU-JMGT1-45 as well as its companion strain, DMKU-JMGT4-14, due to the 53 and 87 nucleotide substitutions in the D1/D2 domains of the LSU rRNA gene and ITS region, respectively, when compared to the closest species.

### 3.3. Molecular Genetic Characterization

**Figure 4:**
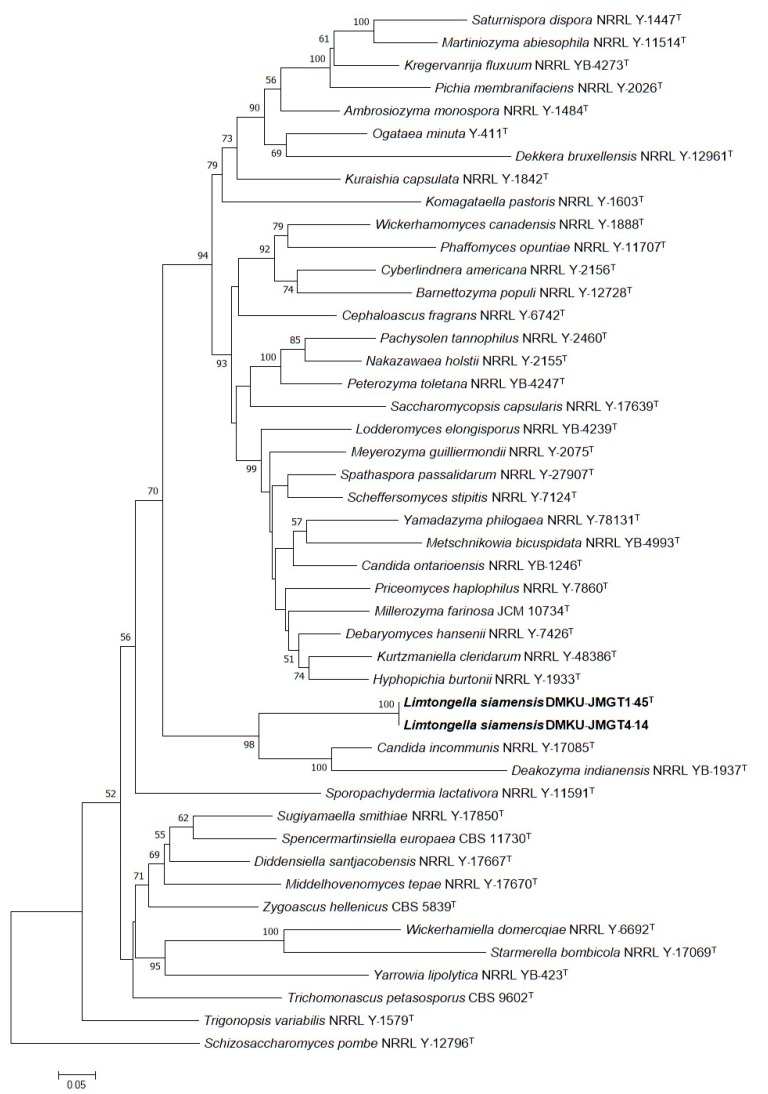
Phylogenetic tree based on maximum likelihood (GTR model) analysis of the small subunit rRNA (SSU), the D1/D2 domain of the large subunit (LSU) rRNA gene, elongation factor-1α (EF-1α), RNA polymerase subunits 1 (RPB1) and 2 (RPB2) sequences showing the taxonomic placement of *Limtongella siamensis* DMKU-JMGT1-45^T^ and its sister species (both are shown in bold letters) within the family Trichomonasceae and related genera in the order Saccharomycetales. The percentage of replicate trees in which the associated taxa clustered together in the bootstrap test (1000 replicates) are shown next to the branches (50%). *Schizosaccharomyces pombe* CBS 356^T^ served as the outgroup species. T, type strain.

According to the criterion proposed by [39], the yeast strain showed greater than 1% nucleotide substitutions of the D1/D2 domain of the LSU rRNA gene, compared with the type strains of species representing a distinguished species (the multiple alignment of the D1/D2 domain of the large subunit (LSU) rRNA gene, SSU, RPB I, RPB II and EF-1α, and genes of the strains DMKU-JMGT1-45^T^, DMKU-JMGT4-14, *C. incommunis* and *Deakozyma indianesis* are shown in Figures S1–S5, respectively). Meanwhile, according to the guidelines proposed by [40], the discrimination of current yeast genera possessing similarity thresholds of lower than 96.31% for ITS and 97.11% for LSU are taken to be different taxa. Based on the analysis of sequence similarity of the D1/D2 domain of the LSU rRNA gene and the ITS region, the strains DMKU-JMGT1-45^T^ and DMKU-JMGT4-14 differ from their closest species, *Candida incommunis* CBS 5604^T^, by 10.41% nucleotide substitutions (53 nucleotide substitutions and 25 gaps of 509 nucleotides) and 20.96% nucleotide substitutions (87 nucleotide substitutions and 46 gaps of 415 nucleotides) of the ITS region. This result revealed that the strains DMKU-JMGT1-45^T^ and DMKU-JMGT4-14 are clearly distinguished from their closest species. In order to study the phylogenetic placement of these strains, a phylogenetic tree was constructed. Since the sequences of these yeast were quite different from other yeasts, the information on the D1/D2 domain of the LSU rRNA gene alone may not be enough to clearly classify the position of the strains. Therefore, phylogenetic tree analysis was constructed by using SSU, the D1/D2 domain of the LSU rRNA gene, EF-1α, and the RNA polymerase subunits 1 (RPB1) and 2 (RPB2) genes of type species from their related species. The accession numbers of the proposing strain and reference strains are shown in Table S2. A phylogenetic analysis based on five genes revealed that the two strains formed a distinguished cluster that was separated from *C. incommunis* CBS 5604^T^ and *D. indianensis* CBS 12903^T^ with high bootstrap support (Figure 4).

### 3.4. Phenotypic Characterization

The phenotype characteristics of *L. siamensis* DMKU-JMGT1-45^T^ and *L. siamensis* DMKU-JMGT4-14 were identical, while the main difference between *L. siamensis* DMKU-JMGT1-45^T^ and *C. incommunis* and *D. indianensis* was its ability to assimilate melibiose, raffinose, inulin, starch, L-arabinose, D-ribose and methanol as a carbon source, as well as in its carbohydrate fermentation, acid production, and its ability to grow in a medium supplemented with cycloheximide. The strains DMKU-JMGT1-45^T^ and DMKU-JMGT4-14 showed positive results in glucose, maltose and trehalose fermentation, positive results in acid production, and negative results in their ability to grow in a medium supplemented with cycloheximide, whereas *C. incommunis* CBS 5604^T^ showed negative, negative, and positive results, respectively (Table 3). The spore formation of these three species is unknown. According to the results of DNA sequence analysis and phenotypic data, the strains DMKU-JMGT1-45^T^ and DMKU-JMGT4-14 were identical and could not be assigned to any currently recognized genera. Consistent with this, we propose the genus *Limtongella* gen. nov. to accommodate members of the clade.

### 3.5. Description of gen. nov.

*Limtongella* (Lim.tong.el’la. L. nom. f. n. *Limtongella* is named in honor of Professor Savitree Limtong for her outstanding studies of yeast systematics in Thailand).

Cells are ovoid and cell division is by multilateral budding. Pseudohyphae are produced. Ascospore formation has not been observed in individual or mixed cultures. Carbohydrate may or may not be fermented. Diazonium blue B (DBB) and urease are negative. Starch-like compounds are not produced.

Phylogenetic placement: Saccharomycetales, Saccharomycotina, Ascomycota.

The type species of the genus is *Limtongella* *siamensis.*

MycoBank accession number is MB832635.

### 3.6. Description of sp. nov.

*Limtongellasiamensis (*si.a.men’sis.N.L. fem.adj. siamensis of or belonging to Siam, the old name of Thailand, where the type strain was isolated).

**Figure 5:**
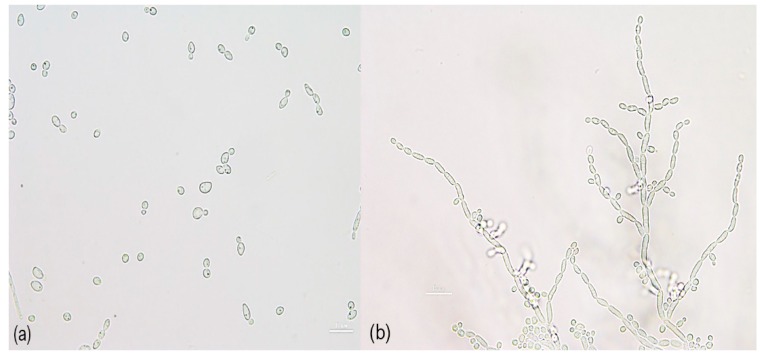
Morphology of *Limtongella siamensis* nov. (DMKU-JMGT1-45^T^): (a) budding cells in a YM broth after three days at 25 °C; (**b**) pseudohyphae formed on PDA after three days at 25 °C. Bars, 10 µm.

Yeast cells, after three days of cultivation on YM agar, are ovoidal and divided by multilateral budding. After three days incubation on YM agar at 25 °C, colonies are white, smooth, butyrous and glistening to dull surface. Ascospore are not formed in individual or mixed culture on any test medium. Pseudohyphae are observed on PDA at 25 °C after three days (Figure 5). Growth occurs at 15, 25, 30 and 35 °C on YM broth. Fermentation of sugars is absent. Glucose, galactose, sorbose, cellobiose, lactose (slow), maltose, melibiose (slow), sucrose, trehalose, melizitose, raffinose (slow), inulin (slow), starch (slow), d-arabinose, l-arabinose, d-ribose (slow), l-rhamnose (weak), d-xylose, galactitol (slow), d-glucitol, inositol, d-mannitol, glycerol, ribitol, ethanol, methanol (slow), citric acid, lactic acid (slow), succinic acid (slow), d-gluconic acid, d-glucoronic acid (slow), galacturonic acid (slow), α-methyl-d-Glucoside, salicin, N-acetyl-d-glucosamine, d-glucono-5-lactone, 2-keto-d-gluconate and 5-keto-d-gluconate are assimilated as sole carbon sources; ammonium sulfate, potassium nitrate, sodium nitrite, ethylamine HCl, l-lysine and cadaverine are assimilated as sole nitrogen sources. Growth in a vitamin-free medium is negative. Growth is not observed in media containing 50% glucose, 60% glucose, 10% NaCl plus 5% glucose and 16% NaCl plus 5% glucose. No growth occurs in a medium supplemented with 0.01 and 0.1% of cycloheximide. Acid production is positive, whereas urease activity and diazonium Blue B reaction are negative.

## 4. Conclusions

In the present study, we have described yeast communities in grease traps, which are high lipid-accumulating habitats. Our results showed that ascomycetous yeast was the dominant yeast and *Candida tropicalis* was the most common yeast found in the grease trap. In addition, 61 yeast isolates from the total showed lipase activity index (LI) values of between 1.1 and 5.6, and the highest LI value belonged to the strain DMKU-JMGT1-45^T^. After quantitative analysis in a liquid medium, the strain DMKU-JMGT1-45^T^ showed the ability to produce lipase, even in a medium without oil supplementation. We also found lipid accumulation inside the cells of this strain. The main fatty acid found in strain DMKU-JMGT1-45^T^ was a C18:1, which is one of the common biodiesel components. These data are preliminary information for lipase production and lipid accumulation studies of this strain for future work to investigate whether it can be applied in the industry. Surprisingly, the strain DMKU-JMGT1-45^T^ showed a significant difference in the D1/D2 domain of the large subunit (LSU) rRNA gene to other described yeasts in GenBank. Hence, we propose the genus *Limtongella* gen. nov. to accommodate the novel species *Limtongella siamensis*. These results suggest that a grease trap is a good source to study yeast communities as well as to discover a potent lipase-producing yeast, which is an important enzyme in industrial applications.

## Figures and Tables

**Table 1:** Summary of described yeast discovered in grease traps.

Yeast Taxa	GenBank Accession Number (D1/D2 Domain)	No. of Yeast Isolates
**Ascomycota**		
**Saccharomycotina**		
**Debaryomycetaceae**		
*Candida aaseri*	NG_054858	16
*Candida metapsilosis*	NG_054815	2
*Candida natalensis*	NG_055225	4
*Candida neustonensis*	NG_054803	1
*Candida orthopsilosis*	NG_054816	6
*Candida parapsilosis*	U45754	2
*Candida tropicalis*	NG_054834	31
*Lodderomyces elongisporus*	U45763	1
*Meyerozyma guilliermondii*	U45709	1
*Schwanniomyces etchellsii*	U45809	2
**Dipodascaceae**		
*Magnusiomyces ingens*	U40127	3
**insertae sedis**		
*Candida rugosa*	FJ768915	3
**Metschnikowiaceae**		
*Candida pseudointermedia*	NG_055407	1
*Kodamaea ohmeri*	NG_042527	1
**Phaffomycetaceae**		
*Candida mengyuniae*	EU043158	12
*Cyberlindnera jadinii*	EF550309	2
**Pichiaceae**		
*Allodekkera sacchari*	AB698502	1
*Candida boidinii*	U70242	1
*Candida ethanolica*	U71073	5
*Candida cylindracea*	NG_055155	7
*Candida pseudolambica*	U71063	7
*Candida sorboxylosa*	U62314	1
*Pichia kudriavzevii*	NG_055104	4
*Pichia manshurica*	NG_055078	4
*Pichia occidentalis*	NG_055110	2
**Saccharomycetaceae**		
*Candida glabrata*	AY198398	1
*Kazachstania humilis*	KY106507	5
*Saccharomyces cerevisiae*	NG_042623	2
**Trichomonasaceae**		
*Candida suthepensis*	AB565751	2
*Wickerhamiella pararugosa*	NG_055327	2
*Wickerhamiella infanticola*	KY106515	11
*Zygoascus bituminiphila*	NG_055308	1
*Zygoascus polysorbophila*	DQ438188	3
**Pezizomycotina**		
**Saccotheciaceae**		
*Aureobasidium melanogenum*	FJ150926	1
**Herpotrichiellaceae**		
*Exophiala dermatitidis*	KF928508	1
**Basidiomycota**		
**Sporidiobolaceae**		
*Rhodotorula mucilaginosa*	NG_055716	1

**Table 2:** The fatty acid contents in *Limtongella siamensis* DMKU-JMGT1-45 and other oleaginous yeasts.

Yeast Species	Fatty Acid (% of Total Fatty Acid)					
	C16:0	C16:1	C18:0	C18:1	C18:2	C18:3
*Limtongella siamensis* DMKU-JMGT1-45	15	28	1.1	40	2	nd
*Cryptococcus albidus*	12	1	3	73	12	nd
*Rhodotorula glutinis*	37	1	3	47	8	nd
*Rhodotorula graminis*	30	2	12	36	15	4
*Yarrowia lipolytica*	11	6	1	28	51	1

nd, not detectable; C16:0, palmitic acid; C16:1, palmitoleic acid; C18:0, stearic acid; C18:1, oleic acid; C18:2, linoleic acid; and C18:3, linolenic acid.

**Table 3:** Differential characteristic between *Limtongella siamensis* DMKU-JMGT1-45^T^, *Candida incommunis* CBS 5604^T^ and *Deakozyma indianensis* CBS 12903^T^. Species: 1, *Limtongella siamensis* DMKU-JMGT1-45^T^ gen. nov., sp. nov.; 2, *Candida incommunis* CBS 5604^T^ data from [32]; and 3, *Deakozyma indianensis* CBS 12903^T^ data from [18].

Characteristics	1	2	3
**Assimilation of carbon compounds**			
**Melibiose**	+	−	−
**Raffinose**	+	−	−
**Inulin**	+	−	−
**Starch**	+	−	−
**L-arabinose**	+	−	−
**D-ribose**	+	−	−
**Methanol**	+	−	−

Abbreviations: +, positive; w, weakly positive; s, slowly positive; v, variable; −, negative.

## Supplementary Materials

The following are available online at https://www.mdpi.com/2076-2607/8/1/27/s1.

## References

[1] Poomtien J., Jindamorakot S., Limtong S., Pinphanichakarn P., Thaniyavarn J. (2013). Two new anamorphic yeasts species, *Cyberlindnera samutprakarnensis* sp. nov. and *Candida thasaenensis* sp. nov., isolated from industrial wastes in Thailand. Antonie Leeuwenhoek.

[2] Jutakanoke R., Endoh R., Takashima M., Ohkuma M., Tanasupawat S., Akaracharanya A. (2017). *Allodekkera sacchari* gen. nov., sp. nov., a yeast species in the Saccharomycetales isolated from a sugar factory. Microbiol. Soc..

[3] Jyoti V., Avneet K. (2006). Yeast lipases: Enzyme purification, biochemical properties and gene cloning. Electron. J. Biotechnol..

[4] Kim H.R., Kim I.H., Hou C.T., Kwon K.I., Shin B.S. (2010). Production of a novel cold-active lipase from *Pichia lynferdii* Y-7723. J. Agric. Food Chem..

[5] Papagora C., Roukas T., Kotzekidou P. (2013). Optimization of extracellular lipase production by *Debaryomyces hansenii* isolates from dry-salted olives using response surface methodology. Food Bioprod. Process..

[6] Boontham W., Angchuan J., Boonmak C., Srisuk N. (2019). *Limtongozyma siamensis* gen. nov., sp. nov., a yeast species in the Saccharomycetales and reassignment of *Candida cylindracea* to the genus *Limtongozyma*. Int. J. Syst. Evol. Microbiol..

[7] Kamini N.R., Fujii T., Kurosu T., Iefuji H. (2000). Production, purification and characterization of an extracellular lipase from the yeast, *Cryptococcus* sp. S-2. Process Biochem..

[8] Ayadi I., Belghith H., Gargouri A., Guerfali M. (2018). Screening of new oleaginous yeasts for single cell oil production, hydrolytic potential exploitation and agro-industrial by-products valorization. Process Saf. Environ. Prot..

[9] Amaretti A., Raimondia S., Leonardia A., Rossia M. (2012). *Candida freyschussii*: An oleaginous yeast producing lipids from glycerol. Chem. Eng. Trans..

[10] Beopoulos A., Nicaud J.M., Gaillardin C. (2011). An overview of lipid metabolism in yeasts and its impact on biotechnological processes. Appl. Microbiol. Biotechnol..

[11] Najjar A., Robert S., Guerin C., Violet-Asther M., Carriere F. (2011). Quantitative study of lipase secretion, extracellular lipolysis, and lipid storage in the yeast *Yarrowia lipolytica* grown in the presence of olive oil: Analogies with lipolysis in humans. Appl. Microbiol. Biotechnol..

[12] Papanikolaou S., Aggelis G. (2011). Lipids of oleaginous yeasts. Part I: Biochemistry of single cell oil production. Eur. J. Lipid Sci. Technol..

[13] Patel A., Arora N., Mehtani J., Pruthi V., Pruthi P.A. (2017). Assessment of fuel properties on the basis of fatty acid profiles of oleaginous yeast for potential biodiesel production. Renew. Sustain. Energy Rev..

[14] Lin C.-Y., Lin Y.-W. (2012). Fuel Characteristics of Biodiesel Produced from a High-Acid Oil from Soybean Soapstock by Supercritical-Methanol Transesterification. Energies.

[15] Li Q., Du W., Liu D. (2008). Perspectives of microbial oils for biodiesel production. Appl. Microbiol. Biotechnol..

[16] Wijayawardene N.N., Hyde K.D., Lumbsch H.T., Liu J.K., Maharachchikumbura S.S.N., Ekanayaka A.H., Tian Q., Phookamsak R. (2018). Outline of Ascomycota: 2017. Fungal Divers..

[17] Klopotek A.V. (1967). *Blastobotrys nivea* gen.nov., sp.nov. Arch. Microbiol..

[18] Kurtzman C.P., Robnett C.J. (2014). Three new anascosporic genera of the Saccharomycotina: *Danielozyma* gen. nov., *Deakozyma* gen. nov. and *Middelhovenomyces* gen. nov. Antonie Leeuwenhoek.

[19] Peter G., Dlauchy D., Price N.P., Kurtzman C.P. (2012). *Diddensiella caesifluorescens* gen. nov., sp. nov., a riboflavin-producing yeast species of the family Trichomonascaceae. Int. J. Syst. Evol. Microbiol..

[20] Kurtzman C.P. (2016). Description of *Groenewaldozyma* gen. nov. for placement of *Candida auringiensis*, *Candida salmanticensis* and *Candida tartarivorans*. Antonie Leeuwenhoek.

[21] Sydow H. (1912). Referate und kritische Besprechungen. Ann. Mycol..

[22] Peter G., Dlauchy D., Tornai-Lehoczk J., Suzuki M., Kurtzman C. (2011). *Spencermartinsiella europaea* gen. nov., sp. nov., a new member of the family Trichomonascaceae. Int. J. Syst. Evol. Microbiol..

[23] Rosa C.A., Lachance M. (1998). The yeast genus *Starmerella* gen. nov. and *Starmerella bombicola* sp. nov., the teleomorph of *Candida bombicola* (Spencer, Gorin & Tullock) Meyer & Yarrow. Int. J. Syst. Evol. Microbiol..

[24] Kurtzman C.P., Robnett C.J. (2007). Multigene phylogenetic analysis of the *Trichomonascus*, *Wickerhamiella* and *Zygoascus* yeast clades, and the proposal of *Sugiyamaella* gen. nov. and 14 new species combinations. FEMS Yeast Res..

[25] Van der Walt J.P., Liebenberg N.V. (1973). The yeast genus *Wickerhamiella* gen. nov. (Ascomycetes). Antonie Leeuwenhoek.

[26] Van der Walt J., Liebenberg N.V. (1980). The yeast genus *Yarrowia* gen. nov. Antonie Leeuwenhoek.

[27] Smith M. (1986). *Zygoascus hellenicus* gen. nov., sp. nov., the teleomorph of *Candida hellenica* (= *C. inositophila* = *C. steatolytica*). Antonie Leeuwenhoek.

[28] Ali S., Rafi H., Ikram ul H. (2010). Production of an extracellular lipase from *Candida lipolytica* and parameter significance analysis by Plackett-Burman design. Eng. Life Sci..

[29] Kraisintu P., Yongmanitchai W., Limtong S. (2010). Selection and optimization for lipid production of a newly isolated oleaginous yeast, *Rhodosporidium toruloides* DMKU3-TK16. Kasetsart J..

[30] Yarrow D. (1998). Methods for the isolation, maintenance and identification of yeasts. Yeasts.

[31] Fowell R.R. (1952). Sodium acetate agar as a sporulation medium for yeasts. Nature.

[32] Lachance M., Boekhout T., Scorzetti G., Fell J.W., Kurtzman C.P. (1998). *Candida* Berkhout. Yeasts.

[33] Rosa C.A., Lachance M.A., Teixeira L.C., Pimenta R.S., Morais P.B. (2007). *Metschnikowia cerradonensis* sp. nov., a yeast species isolated from ephemeral flowers and their nitidulid beetles in Brazil. Int. J. Syst. Evol. Microbiol..

[34] Kurtzman C., Robnett C. (2003). Phylogenetic relationships among yeasts of the complex determined from multigene sequence analyses. FEMS Yeast Res..

[35] Hall T.A. (1999). BioEdit: A user-friendly biological sequence alignment editor and analysis program for Windows 95/98/NT. Nucleic Acids Symp. Ser..

[36] Kumar S., Stecher G., Tamura K. (2016). MEGA7: Molecular Evolutionary Genetics Analysis version 7.0 for bigger datasets. Mol. Biol. Evol..

[37] Felsenstein J. (1985). Confidence limits on phylogenies: An approach using the bootstrap. Evolution.

[38] Duarte S.H., Ansolin M., Maugeri F. (2014). Cultivation of *Candida* sp. LEB-M3 in glycerol: Lipid accumulation and prediction of biodiesel quality parameters. Bioresour. Technol..

[39] Kurtzman C.P., Robnett C.J. (1998). Identification and phylogeny of ascomycetous yeasts from analysis of nuclear large subunit (26S) ribosomal DNA partial sequences. Antonie Leeuwenhoek.

[40] Vu D., Groenewald M., Szoke S., Cardinali G., Eberhardt U., Stielow B., de Vries M., Verkleij G.J., Crous P.W., Boekhout T. (2016). DNA barcoding analysis of more than 9000 yeast isolates contributes to quantitative thresholds for yeast species and genera delimitation. Stud. Mycol..

